# Application of a mathematical model for ergonomics in lean manufacturing

**DOI:** 10.1016/j.dib.2017.06.050

**Published:** 2017-07-04

**Authors:** Lucia Botti, Cristina Mora, Alberto Regattieri

**Affiliations:** Department of Industrial Engineering, University of Bologna, Viale del Risorgimento, 2, 40136 Bologna, Italy

## Abstract

The data presented in this article are related to the research article “Integrating ergonomics and lean manufacturing principles in a hybrid assembly line” (Botti et al., 2017) [1]. The results refer to the application of the mathematical model for the design of lean processes in hybrid assembly lines, meeting both the lean principles and the ergonomic requirements for safe assembly work. Data show that the success of a lean strategy is possible when ergonomics of workers is a parameter of the assembly process design.

## Specifications Table

**Table:** 

Subject area	*Industrial engineering*
More specific subject area	*Human factors, industrial and logistic system design*
Type of data	*Tables, Figures*
How data was acquired	*Input data acquired on field during the research project*
Data format	*Raw and analyzed data*
Experimental factors	*Data acquired during the daily activity of assembly workers at the reference manufacturing company*
Experimental features	*Data acquired during the daily activity of assembly workers at the reference manufacturing company*
Data source location	*Not applicable for confidentiality reasons*
Data accessibility	*Data in this article and in the related research article*[1].

## **Value of the data**

• The input data, i.e. parameter values, may be exported in order to be used by different mathematical models.
• The output data may be used to define different decision functions for the choice of the optimal solution among the Pareto points.
• The output data, e.g variable values, may be exported in order to compare them with other results after the application of input data to different models.

## 1 Data

The model inputs refer to a manual assembly line with 6 manual workstations and 6 manual workers. A single worker is assigned to each manual workstation. The assembly task sequence is the same for each product type. Each task is standardizable and the assembly activities are not complex. Sensitive values of the manual assembly-process parameters are hidden, e.g. cycle times, takt times and batch sizes, for confidentiality reasons. The safety time varies from 1 to 3 h while the mean lateness of manual workstations varies from 2 to 12 s, depending on the product type and the task. The following Table 1 shows the other model parameters and the OCRA parameters for the ergonomic risk assessment through the OCRA method [2], [3].

**Table 1:** Parameters of the mathematical model.

t	*1*	*2*	*3*	*4*	*5*	*6*
i [machines]	1	1	1	1	1	1
$l_{\max t}$ [workers]	1	1	1	1	1	1
$o_{t}$ [%]	2	2	1	2	1	2
${o\prime }_{t}$ [%]	4	5	3	5	3	5
$q_{t}$ [€/h]	2.80	2.40	1.20	1.20	1.20	2.40
$r_{t}$ [€/machine and hour]	100	100	100	100	100	100
$x_{t}$ [€/h and machine]	56	40.88	32.1	47.44	32.1	40.88
$y_{t}$ [€/h and worker]	15	15	15	15	15	15
*OCRA parameters*						
$n_{\begin{array}{c} \mathrm{TC},t \end{array}}$*Product 1*	14	8	5	4	3	12
$n_{\begin{array}{c} \mathrm{TC},t \end{array}}$*Product 2*	15	8	5	5	3	12
$n_{\begin{array}{c} \mathrm{TC},t \end{array}}$*Product 3*	14	8	5	4	3	12
$n_{\begin{array}{c} \mathrm{TC},t \end{array}}$*Product 4*	15	10	5	5	3	12
$k_{f}$*Product from 1 to 4*	30	30	30	30	30	30
$F_{M,t}$*Product from 1 to 4*	0.65	0.35	1.00	0.85	1.00	0.20
$P_{M,t}$*Product from 1 to 4*	0.60	0.60	1.00	0.60	1.00	0.60
ReM,t*Product from 1 to 4*	1.00	0.70	1.00	0.70	1.00	0.70
$A_{M,t}$*Product from 1 to 4*	1.00	0.90	1.00	0.95	1.00	0.80
${\mathrm{Rc}}_{M}$	0.60	0.60	0.60	0.60	0.60	0.60
$t_{M}$	1.00	1.00	1.00	1.00	1.00	1.00

Particularly, the values of the technical actions refer to the most stressed arm, for each worker. The work shift is of 8 h. A lunch break and two breaks of 10 min each are distributed among the 8-h shift. Job rotations are not allowed during the work shift and each worker performs the same single task for the whole 8 h. As a consequence, repetitive manual tasks last for a relevant part of the shift. The OCRA indices in Table 2 define the workers exposure to repetitive movements of the upper limbs.

**Table 2:** OCRA index for each worker.

*Worker*	*Task*	*OCRA index*
Worker 1	1	3.4
Worker 2	2	1.3
Worker 3	3	0.7
Worker 4	4	1.5
Worker 5	5	0.6
Worker 6	6	3.7

## 2 Experimental design, materials and methods

The introduced data define the model inputs for the considered case study. 48 binary variables are introduced subjected to 60 feasibility constraints. The model and the input data are coded in AMPL language and processed adopting Gurobi Optimizer© v.5.5 solver. An Intel® CoreTM i7-4770 CPU @ 3.50 GHz and 32.0GB RAM workstation is used. The average solving time is approximately of 0.5 s.

**Fig. 1:**
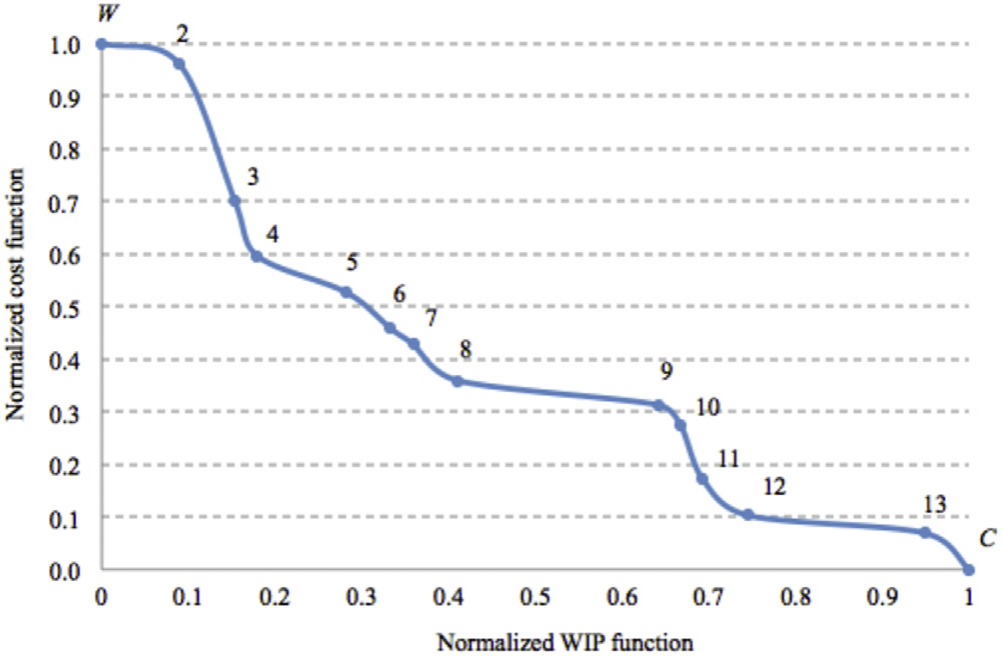
Normalized Pareto frontier [1].

The Normalized Pareto frontier in Fig. 1 shows the trends of the two objective functions in the normalized *WIP-Cost* diagram [4]. Particularly, the points from *W* to *C* are the Pareto points composing the normalized Pareto frontier (Fig. 1). Each Pareto point represents an effective non-dominated trade-off assembly layout configuration.

The following Table 3 shows the coordinates of each Pareto point.

**Table 3:** Value of the normalized functions for each Pareto point.

Pareto point	Normalized WIP function	Normalized cost function
*W*	0.00	1.00
2	0.09	0.96
3	0.15	0.70
4	0.18	0.60
5	0.28	0.53
6	0.33	0.46
7	0.36	0.43
8	0.41	0.36
9	0.64	0.31
10	0.67	0.28
11	0.69	0.17
12	0.74	0.10
13	0.95	0.07
*C*	1.00	0.00

**Fig. 2:**
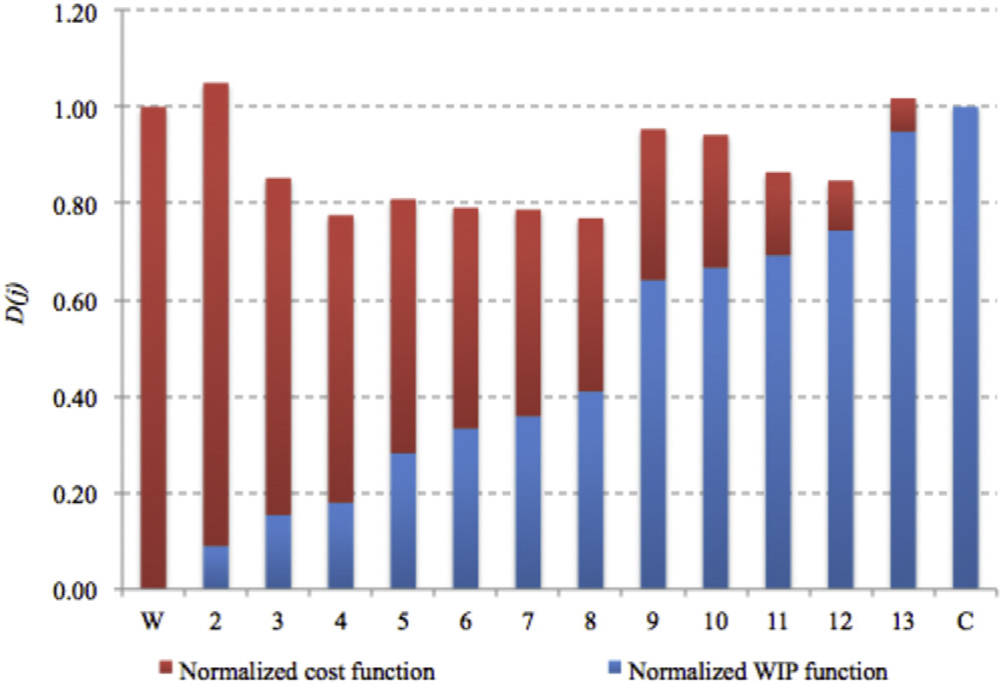
Values of decision function *D*(*j*) for each Pareto point [1].

The following Fig. 2 and Table 3 show the values of decision function *D(j)* for each Pareto point Table 4.

**Table 4:** Decision function value for each Pareto point.

*j*	*W*	*2*	*3*	*4*	*5*	*6*	*7*	*8*	*9*	*10*	*11*	*12*	*13*	*C*
*D* (*j*)	1.00	1.05	0.85	0.78	0.81	0.79	0.79	0.77	0.95	0.94	0.86	0.85	1.02	1.00

**Fig. 3:**
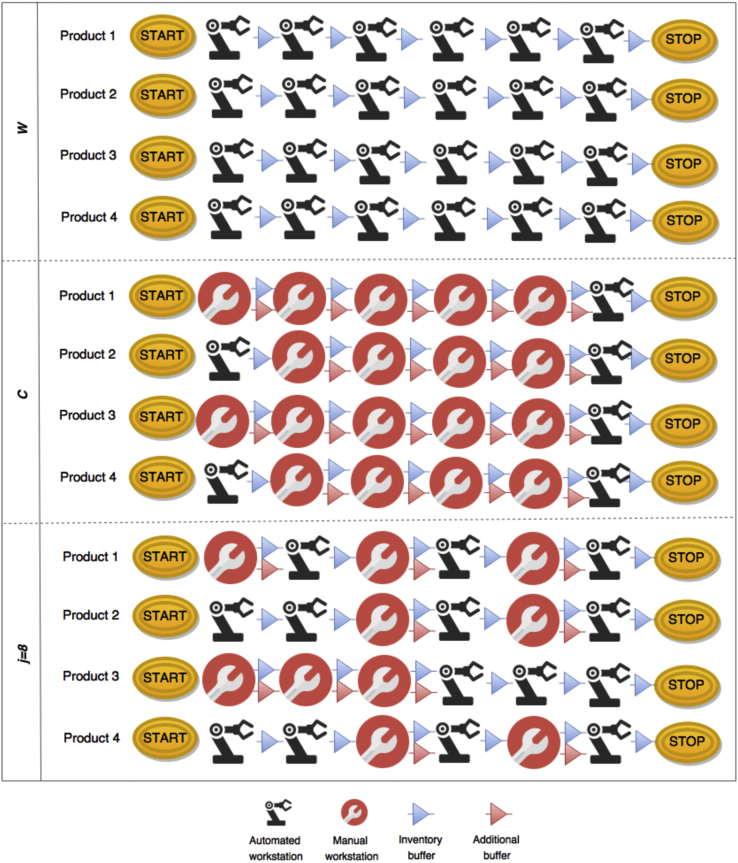
Assembly layouts for solutions in points *W, C* and *j*=8 [1].

The solution in point *j*=8 minimises the decision function *D(j)*. The following Fig. 3 shows the assembly layouts for solutions in points *W, C* and *j*=8.
